# Decoding Occult Spinal Dysraphism: A Pictorial Essay of Split Cord Malformation With Prenatal and Postmortem Imaging Correlation

**DOI:** 10.1055/a-2844-6196

**Published:** 2026-04-13

**Authors:** Kakoly Borthakur, Nishigandha Mali, Ishika Borthakur

**Affiliations:** 1 Women's Imaging, Aarti Diagnostic Centre, Navi Mumbai, India

## Introduction

Split cord malformation (SCM) is a rare congenital anomaly within the spectrum of occult spinal dysraphisms (OSDs), involving longitudinal splitting of the spinal cord by a fibroosseous or fibrous septum. It is frequently associated with tethered cord syndrome, vertebral anomalies, lipomas, and dorsal dermal sinuses. Pang et al. classified SCM into Type I (diastematomyelia) and Type II (diplomyelia) based on the septal composition and dural sac configuration. Although SCM accounts for ~5% of congenital spinal defects, antenatal diagnosis is rare. We present a mid-trimester prenatal case of SCM with tethered cord, posterior lipoma, vertebral anomalies, dorsal dermal sinus, and unilateral club foot, diagnosed on ultrasound and confirmed postmortem. Multiplanar high-resolution ultrasound revealed a widened spinal canal, segmental cord splitting, low-lying conus, and a fat-intense lesion abutting the lamina and extending into the subcutaneous plane. Postmortem imaging confirmed these findings, including dural calcification and vertebral anomalies.

**Fig. 1:**
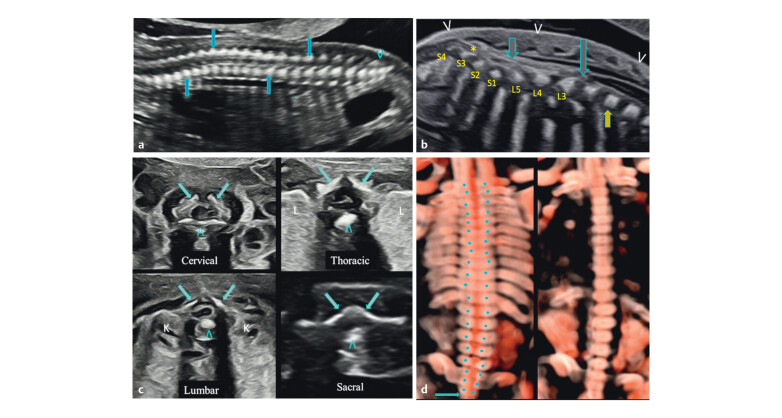
Mid trimester sections of the normal spine: ( **a** ) A paramedian sagittal section shows the posterior ossific centres (downward arrow), anterior ossific centres (upward arrow), and two rows converging to form the sacral taper with dorsal uptilt (arrowhead). ( **b** ) A midsagittal section of the normal lumbosacral spine—only the anterior ossific centre row is seen (solid arrows), conus medullaris (long arrow), cauda equina (short arrow), and filum terminale (*). Conus medullaris is at the lower border of L3. Counting is done from S4 cephalad or from T12 caudal. Intact skin layers seen over the entire length of the spine (arrowheads). ( **c** ) Axial sections of the cervical, thoracic, lumbar and sacral spine—two posterior ossific centres (arrows), one anterior ossific centre (arrowhead), lungs (L), kidneys (K), and spinal cord is seen in the vertebral canal as an ovoid hypoechoic structure fillied with CSF and a central echogenic dot or an oval structure corresponding to the cord, except at the sacral level where the cord with cauda equina is completely echogenic. ( **d** ) Coronal sections, the left posterior section showing the posterior ossific centres forming two parallel rows (rail road appearance) converging at the sacral tip (arrow) and the right anterior section showing the anterior ossific centres as a single row.

Understanding normal fetal spine anatomy is essential for identifying subtle deviations. In the mid-trimester, the spine appears as two echogenic lines in sagittal views, representing anterior and posterior ossific centres. The spinal cord is seen as a hypoechoic structure with a central echogenic canal, with the conus typically terminating at L2–L3 ( Fig. 1a and b ). Axial views show one anterior and two posterior ossific centres enclosing the vertebral canal, which contains the cord as a hypoechoic circle with a central dot ( Fig. 1c ). In ventral coronal sections, anterior centres appear ventrally, while posterior centres form two parallel rows converging toward the sacral tip ( Fig. 1d ).

## Case description

**Fig. 2:**
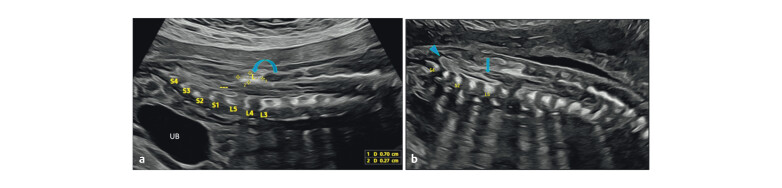
The midsagittal section of the lumbosacral spine. ( **a** ) An echogenic focus noted dorsal to the spinal canal at L3–L5 level (curved arrow). ( **b** ) Conus medullaris terminating below the L5 level (straight arrow), the central cord noted at the conus as a thin echogenic line bordered by CSF and dural linings, and a short thick blunted filum terminale noted (arrowhead).

**Fig. 3:**
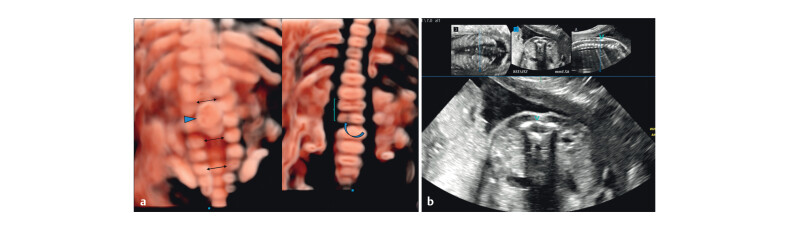
( **a** ) Coronal sections of the lumbosacral spine shows a widened spinal canal with divergent posterior ossification centres (arrows) and a well defined soft tissue mass in between L2 and L3 vertebrae (arrowhead); L1 and L2 vertebral bodies are reduced in height suggestive of hypoplasia (brackets) and a cleft in L2 vertebral body (curved arrow). ( **b** ) Multiplanar axial reconstruction at the L4 level shows the echogenic structure in continuity with the posterior ossification structures in the parasagittal view (arrowhead).

**Fig. 4:**
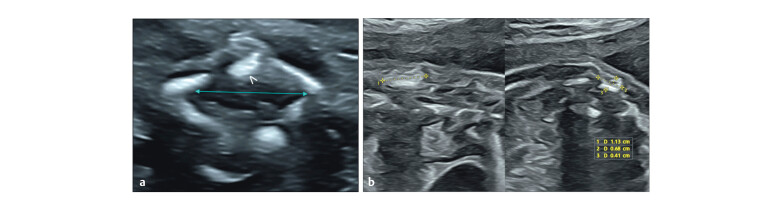
Transverse sections of the lumbosacral spine at L4 level ( **a** ) shows spacing between the rear ossification centres, widened spinal canal (between arrows) and an echogenic non-shadowing focus obliquely placed posterior to the widened spinal canal (arrowhead). ( **b** ) Oblique transverse sections of the at the L4 level shows two hyperechoic lesions within the soft tissues posterior to the spine (between calipers).

**Fig. 5:**
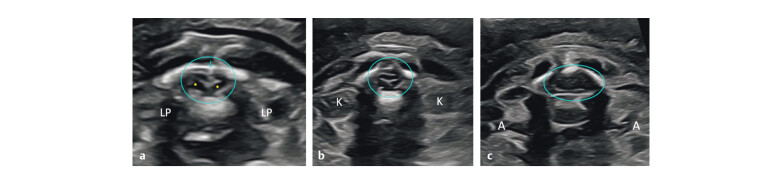
Transverse sections of the cord at the lumbar level from a lower pole of kidneys to the adrenals showing tethering of the cord with splitting. ( **a** ) The fibrous septum (arrow) within the canal anchoring the dura and cord to the posterior bony elements causing tethering and splitting of the dura in two parts (*). ( **b** ) The dural split with a septum anteriorly; an oedematous thickened cord above. LP → lower pole of kidneys; K → mid renal level, A → adrenals.

**Fig. 6:**
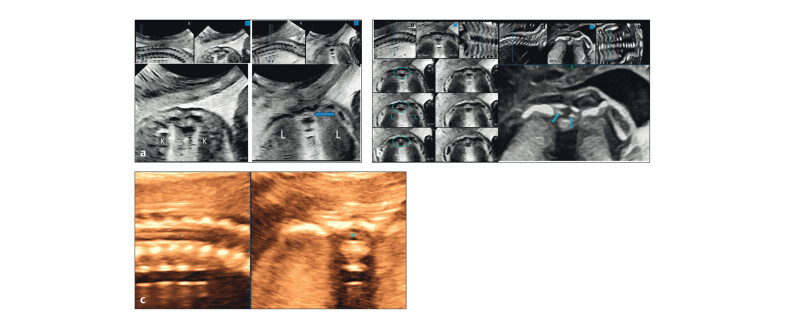
( **a** ) Rendered transverse sections of the cord at upper lumbar and lower dorsal using multislice 0.5 mm slices at 1.5 mm interval shows a thickened oedematous cord at the upper pole of kidneys (K) and a normal calibre cord with no splitting at the level of lung bases (L). Arrows denote the cord. Inverted rendering has been used for better appreciation of the cord. Inset: Slice reference in the sagittal plane. ( **b** ) Transverse sections of the cord in the upper dorsal level using multislice imaging with inverted rendering (left) shows a split cord with a fibrous septum in between (circled) with a thickened oedematous cord in the adjacent slices, Lungs → L. One section shows calcification in the split dura (arrows), confirmed postmortem. ( **c** ) Normal cord at mid thoracic level (*).

**Fig. 7:**
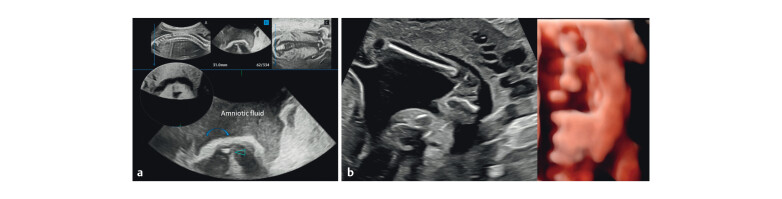
( **a** ) Oblique transverse section of the soft tissues at the lower end of spine (blue lines denoting the rendered image in sagittal and coronal planes) shows a soft tissue tract (arrowhead) extending from the spinal tip towards the skin (curved arrow). The inset shows inverted rendering. ( **b** ) 2D and 3D rendering of the fetal left foot shows unilateral congenital talipes equinovarus deformity (club foot).

A 23-year-old woman (G2 P1) underwent a routine anomaly scan at 22+3 weeks. Early scans including NT at 13.1 weeks were normal, and first trimester biomarkers were not done. In the current scan, biometry and brain structures were normal and no other anomaly seen. An echogenic focus was noted along the posterior lumbar spine (L3–L5), measuring 7×3 mm ( Fig. 2a ). The conus was low-lying at L4–L5 with a short, thick filum terminale with a blunt termination ( Fig. 2b ). Multiplanar imaging showed separation of posterior ossification centres and a soft tissue mass between L2 and L3. L1–L2 vertebrae were hypoplastic, with a cleft in L2 ( Fig. 3a ). The echogenic focus corresponded to a linear structure posterior to the widened canal, continuous with posterior elements ( Fig. 3b ). Dorsal to this, two hyperechoic masses were seen within posterior soft tissues, measuring 11 mm and 7 mm ( Fig. 4a and b ). The canal was widened with thin echogenic septae splitting the cord and oedematous above ( Fig. 5a–c ). Segmental discontinuous splitting was noted at thoracic levels. Dural calcification was seen ( Fig. 6a and b ). An echogenic lesion extended from the terminal spine toward the skin ( Fig. 7a ). The left-sided club foot was present ( Fig. 7b ). No other anomalies were detected.

A provisional diagnosis of closed spinal dysraphism with tethered split cord, dorsal lipoma, vertebral anomalies, and possible dermal sinus was made. The patient was counselled and offered invasive testing with whole exome sequencing (WES). The WES was normal; however, they opted for termination at 23 weeks.

### Postmortem findings

**Fig. 8:**
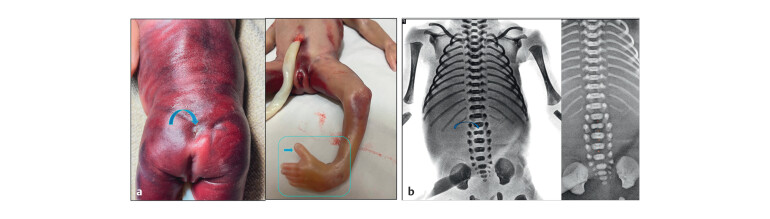
( **a** ) Gross postmortem examination: Cutaneous stigmata noted at the sacral level suggestive of a dorsal dermal sinus (curved Arrow). A grossly normal looking female fetus with left sided club foot (Box) and upright left toe (arrow). ( **b** ) Postmortem radiographs: Widened interpedicular distance from D12 to S2 with separated posterior elements (pedicle and lamina). The L2 vertebral body is reduced in height with a suspicious cleft (curved arrow). The fat density noted in the disc spaces at L1–L5 (Dots) corresponding to the mass on the prenatal coronal image.

**Fig. 9:**
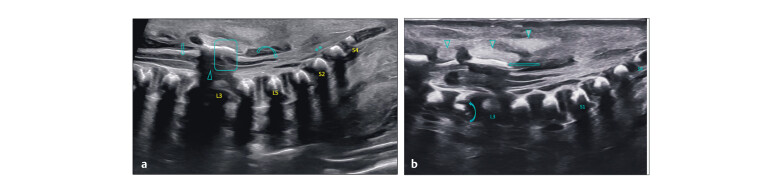
Postmortem ultrasound: ( **a** ) Cord tethered by a septum (box), split at the level of the bony defect at L2–L3 (Arrowhead), oedematous and thickened cord above (arrow). The conus medullaris terminating at the L5 level (curved arrow) and a thick filum terminale (**). ( **b** ) Coronal cleft seen in the anterior ossification centre of L2 (curved Arrow). Fused lamina of L2 and L3 vertebrae noted conforming to the echogenic lesion seen on prenatal imaging (arrow). Dorsal Lipoma noted with extradural and subcutaneous components (arrowheads).

**Fig. 10:**
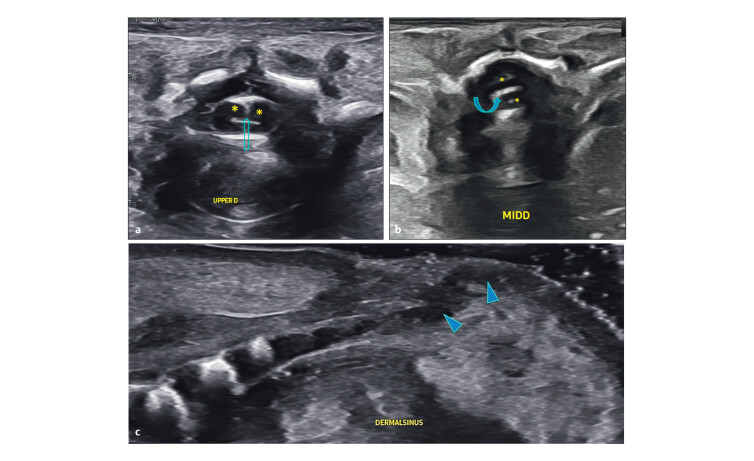
( **a** ) Splitting of the cord into two dural sacs (*) with a sagittal septum (arrow) at the upper dorsal level. ( **b** ) Dural calcification in the mid dorsal level with a coronally oriented fibroosseous septum (curved arrow) with two cords (*). ( **c** ) A hypoechoic tract connecting the tip of the spine with the dorsal dermal sinus (arrowheads).

Gross examination confirmed a female fetus with a left club foot and a dorsal dermal sinus in the lower sacral region ( Fig. 8a ). Autopsy radiographs showed widened interpedicular distance, separation of posterior elements from D12 to S2, and a hypoplastic L2 vertebral body with a cleft ( Fig. 8b ). Autopsy ultrasound confirmed SCM with conus terminating at L5 and a thick filum terminale ( Fig. 9a ). The cord was split at L2–L3 and oedematous above. A coronal cleft was seen in the anterior ossification centre of L2. The dorsal echogenic lesion was confirmed as the fused lamina of L2–L3. A multilobulated fat-intense lesion abutting the lamina extended into the subcutaneous plane, consistent with a posterior lipoma ( Fig. 9b ). Discontinuous nonsegmental splitting at higher levels and a fibroosseous septum at the mid-thoracic level were confirmed ( Fig. 10a and b ). The terminal spine extended to the dermal sinus ( Fig. 10c ).

## Discussion

**Fig. 11:**
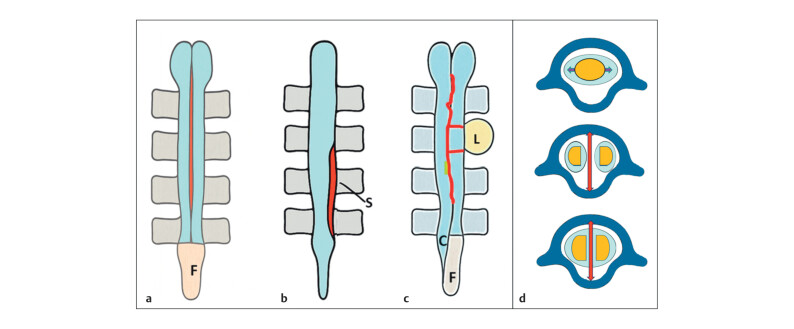
Schematic representation of split cord malformation (SCM): ( **a** ) Type I SCM: Two dural sac split by a bony septum. ( **b** ) Type II SCM: Single dural sac with the cord divided by a fibrous/osseous septum in a single spinal segment. ( **c** ) Type III SCM: Cord split both longitudinally and horizontally at multiple levels, the conus low lying (C), thick filum terminale (F) and a dorsal lipoma (L) as in this case. ( **d** ) Transverse sections: top-normal cord with lateral nerve roots within a single dural sac; middle-Type I SCM with two dural sacs with their own split cord; Bottom- Single dural sac with a split cord. Blue—dural sac; red—Septum splitting the cord; orange—Spinal cord; and yellow—Dorsal lipoma.

SCM arises from defective embryogenesis, resulting in the longitudinal division of the spinal cord. This case suggests a rare composite SCM—alternating split and intact cord segments—unreported in the prenatal imaging literature. SCM accounts for ~3% of OSD, is more common in females, and typically affects the lower thoracic and upper lumbar regions (Arbelo-Pérez et al. 2023). The septum anchors the cord to spinal elements, impeding ascent and causing tethered cord syndrome ( Fig. 11a–d ). Associated bony anomalies include vertebral clefts, absent spinous processes, segmentation defects, and fusion anomalies. Laminar fusion and spina bifida—pathognomonic for SCM—were present in this case (Kleinrok et al., 2025). Depending on the type and extent, SCM may present with neurological symptoms of varying severity. It may be isolated or associated with CNS or systemic anomalies. Muscle weakness and atrophy in one lower limb are seen in 50% of lumbar SCM; the club foot is the most common orthopaedic deformity (Mahapatra et al., 2017).

Prenatal ultrasound can detect SCM during mid-trimester scans when performed systematically (Wei et al., 2017). Transverse sections best depict bony and soft tissue anomalies; longitudinal views assess the cord and conus. While most cases are Type I or II, a composite Type III SCM with multiple, non-contiguous splits has also been described (Vaishya et al., 1001). This case—with postmortem confirmation—is highly suggestive of Type III SCM, although magnetic resonance imaging correlation was unavailable. WES was normal, completing the prenatal work-up. This case illustrates the value of systematic prenatal ultrasound and postmortem correlation in diagnosing SCM and guiding counselling.

